# Common Creatine Kinase gene mutation results in falsely reassuring CK levels in muscle disorders

**DOI:** 10.1093/qjmed/hcv215

**Published:** 2015-11-13

**Authors:** B. Wallace, M.K. Siddiqui, C.N.A. Palmer, J. George

**Affiliations:** From the 1 Division of Cardiovascular & Diabetes Medicine, University of Dundee, Ninewells Hospital & Medical School, Dundee DD1 9SY, UK

Learning point for clinicians

The mean allele frequency of this SNP is 0.02 (∼2.5 million people in the UK are carriers). Therefore, greater awareness of this CKM SNP is needed as there may be instances where the diagnosis of rhabdomyolysis, statin-induced myopathy, poly-/dermatomyositis and other muscle conditions that result in raised CK’s, is missed in these patients.

## Introduction

Creatine Kinase (CK) is the enzyme responsible for catalysing the exchange of phosphates in the creatine/phosphocreatine shuttle.^1^ Skeletal muscle CK is made up of 90% muscle CK (CK-MM) and 10% heart CK (CK-MB).^2^^,^^3^ In muscle damage (e.g. rhabdomyolysis), extreme exercise and statin-induced myositis, serum CK can be raised in excess of 10 times the upper limit of normal.^4^ However, the recent discovery of a single nucleotide polymorphism(SNP), rs11559024 in the *CKM* gene^5^ results in an inability to produce skeletal muscle type CK and therefore low levels of CK in clinical scenarios that may result in crucial diagnosis such as rhabdomyolysis and statin-induced myositis being missed.

## Case report

A female patient was found to be homozygous for rs11559024 with consistently low CK values. The patient had a past history of Type 2 Diabetes Mellitus, Chronic Kidney Disease and Peripheral Vascular Disease.

Following an admission with extensive necrotising fasciitis, she underwent a debridement procedure for extensive necrotising fasciitis. Post-operative blood samples indicated her CK reached a maximum of 22 IU/l [nr 35-175 IU/l] the day after her surgery. Whilst recovering this patient’s CK returned to 8 IU/l within 48 h after the operation.

Further admissions in subsequent years for a hemicolectomy (for bowel cancer) also revealed significant blunting in her expected CK levels post-operatively. Her pre-operative CK of 34 IU/l remained relatively unchanged post-operatively (28 IU/l).

## Discussion

As a carrier of two copies of the rare allele at the SNP rs11559024 of her *CKM* gene, this patient was unable to produce measurable functioning skeletal muscle type CK resulting in consistently low serum CK even following necrotising fasciitis and open surgery. In her past history, the patient was also deemed ‘intolerant’ to statins and was switched multiple times between different statins. Her CK levels were falsely reassuring when checked by clinicians looking for evidence of a statin-induced myositis.

*Conflict of interest:* None declared.

## References

[1] Wyss M Kaddurah-Daouk R Creatine and creatinine metabolism Physiol Rev 2000; 80:1107–213. 10893433 10.1152/physrev.2000.80.3.1107

[2] Wallimann T Tokarska-Schlattner M Schlattner U. The creatine kinase system and pleiotropic effects of creatine. Amino Acids 2011; 40:1271–96. 21448658 10.1007/s00726-011-0877-3 PMC3080659

[3] Baird MF Graham SM Baker JS Bickerstaff GF. Creatine-kinase- and exercise-related muscle damage implications for muscle performance and recovery. J Nutr Metab 2012; 2012:960363 22288008 10.1155/2012/960363 PMC3263635

[4] Gagliano M Corona D Giuffrida G Giaquinta A Tallarita T Zerbo D, Low-intensity body building exercise induced rhabdomyolysis: a case report. Cases J 2009; 2:7 19123934 10.1186/1757-1626-2-7 PMC2628870

[5] Dube M-P Zetler R Barhdadi A Brown A Mongrain I Normand V, CKM and LILRB5 are associated with serum levels of creatine kinase. Circ Cardiovasc Genet 2014; 7:880–6. 25214527 10.1161/CIRCGENETICS.113.000395

